# Cost-Effectiveness of the Third-Agent Class in Treatment-Naive Human Immunodeficiency Virus-Infected Patients in Portugal

**DOI:** 10.1371/journal.pone.0044774

**Published:** 2012-09-17

**Authors:** Filipa Aragão, José Vera, Inês Vaz Pinto

**Affiliations:** 1 Escola Nacional de Saúde Pública, Universidade Nova de Lisboa, Lisboa, Lisboa, Portugal; 2 Associação SER+, Rua André Homem, Cascais, Cascais, Portugal; 3 HPP - Hospital de Cascais, Alcabideche, Cascais, Portugal; University of Ottawa, Canada

## Abstract

**Introduction:**

Current Portuguese HIV treatment guidelines recommend initiating antiretroviral therapy with a regimen composed of two Nucleoside Reverse Transcriptase Inhibitors plus one Non-nucleoside Reverse Transcriptase Inhibitor (2NRTI+NNRTI) or two Nucleoside Reverse Transcriptase Inhibitors plus one boosted protease inhibitor (2NRTI+PI/r). Given the lower daily cost of NNRTI as the third agent when compared to the average daily costs of PI/r, it is relevant to estimate the long term impact of each treatment option in the Portuguese context.

**Methods:**

We developed a microsimulation discrete events model for cost-effectiveness analysis of HIV treatment, simulating individual paths from ART initiation to death. Four driving forces determine the course of events: CD4+ cell count, viral load, resistance and adherence. Distributions of time to event are conditional to individuals’ characteristics and past history. Time to event was modeled using parametric survival analysis using Stata 11®. Disease progression was structured according to therapy lines and the model was parameterized with cohort Portuguese observational data. All resources were valued at 2009 prices. The National Health Service’s perspective was assumed considering a lifetime horizon and a 5% annual discount rate.

**Results:**

In this analysis, initiating therapy with two Nucleoside Reverse Transcriptase Inhibitors plus one Non-nucleoside Reverse Transcriptase Inhibitor reduces the average number of switches by 17%, saves 19.573€ per individual and increases life expectancy by 1.7 months showing to be a dominant strategy in 57% of the simulations when compared to two Nucleoside Reverse Transcriptase Inhibitors plus one boosted protease inhibitor.

**Conclusion:**

This study suggests that, when clinically valid, initiating therapy with two Nucleoside Reverse Transcriptase Inhibitors plus one Non-nucleoside Reverse Transcriptase Inhibitor is a cost-saving strategy and equally effective when compared to two Nucleoside Reverse Transcriptase Inhibitors plus one boosted protease inhibitor as the first regimen.

## Introduction

Human immunodeficiency virus (HIV) infection remains a major public health concern in Europe, with evidence of increasing transmission in several countries. From 2000 to 2009, the rate of newly HIV diagnosed cases reported has almost doubled in the European Region, from 57 to 92 cases per million. On the other hand, the number of acquired immune deficiency syndrome (AIDS) cases has continued to decline, with the exception of eastern countries, where it has increased [1]. Portugal has the tenth highest incidence of HIV infection (99 cases per million) and the sixth highest incidence of AIDS (28 cases per million) over the 53 countries of the European Region [2].

Since 1983, a total of 39,347 cases of HIV infection have been notified in Portugal of whom 23% have died. Within alive, 29% have been diagnosed with AIDS, 11% have developed symptoms and the remaining 60% are in an asymptomatic stage of the infection [3]. Notwithstanding, the real number of HIV infections is unknown and likely to be significantly higher than the diagnosed and notified number of cases [4].

In 2009, antiretroviral drug expenditure in Portugal was estimated in 193.23 million Euros (22,409 HIV individuals on antiretroviral therapy (ART)) [5] and HIV related National Health Service (NHS) hospitalizations in 11.4 million Euros (*Ministério da Saúde, Administração Central do Sistema de Saúde - Inpatient care episodes in the Portuguese National Health Care Service Database)*.

Between 2008 and 2009, pharmaceutical expenditure grew 7% in the NHS hospital market and antiretroviral drugs were a major driver [6], representing about 17% of the pharmaceutical expenditure in the hospital market. In 2009, several cost restriction actions were recommended and implemented due to country’s excessive deficit and within that context antiretroviral drugs’ expenditure has been singled out as a target.

Current Portuguese HIV treatment guidelines [7] recommend initiating treatment with a regimen composed of either two nucleoside analogue reverse transcriptase inhibitors (NRTI) plus one non-nucleoside reverse transcriptase inhibitor (NNRTI) or two NRTI plus boosted protease inhibitor (PI/r) in accordance with several other international guidelines [8]–[13]. However, those were gathered not considering cost-effectiveness evidence.

NNRTI and PI/r are considered clinically equivalent, in the sense that elements of both classes are considered as first choices in clinical recommendations, being differently prescribed according to clinical criteria (integrase inhibitors, a third option, where not widely available during the study period and are therefore not considered in this analysis). The average daily cost of PI/r is significantly higher than that of NNRTIs (*Aragão F. Budget Impact, in terms of antiretroviral costs, of switching patients on a regimen containing boosted protease inhibitors or the components of the single tablet regimen to the single tablet regimen for treatment of HIV-1 Infection. 13rd Conference of the European AIDS Clinical Society, Belgrade, Out 2011. P7.5/5*). Consequently, in a search for a more efficient use of resources, especially given the increasing cost containment pressure in Portugal, it is relevant to compare the effectiveness of each treatment option in routine care setting and to estimate the corresponding long term impact within a cost-effectiveness framework.

## Methods

### The Discrete Events Microsimulation Cost-effectiveness Model (DEMCEM) Overview

In line with models such as the Cost-Effectiveness of Preventing AIDS Complications (CEPAC) model [14], the Antiretroviral Drug Valuation and Cost-Effectiveness (ADVANCE) [15], the AntiRetroviral Analysis by Monte Carlo Individual Simulation (ARAMIS) [16] and the model by Johnston et al. [17], our model is a microsimulation model where individual paths are simulated rather than taking a cohort simulation approach as performed in traditional Markov models. Comparison of the two types of models has been discussed in the literature [18], [19].

Our model differs from other microsimulation models in that we take a discrete event approach. In discrete-event simulation, event occurrences determine time advances and outcomes are updated at the time the event occurs and not at the end of a predetermined time period. Moreover, discrete-event simulation is particularly useful when interaction between individuals is of relevance as is the case with infectious diseases [20]. This type of simulation has been suggested and applied in a variety of settings [21]–[24]. Of particular similarity to our model, is the Birmingham Rheumatoid Arthritis Model (BRAM) [21]. The two models are programmed in TreeAge Pro2009® and follow the same method to determine timing of activities. Our model differs from the former in that we model the parameters of the time to event distributions as a function of patients’ characteristics and history of past events (using parametric survival analysis) instead of assuming that distribution parameters are fixed. Moreover, it obviously relates to a different disease.

**Figure 1:**
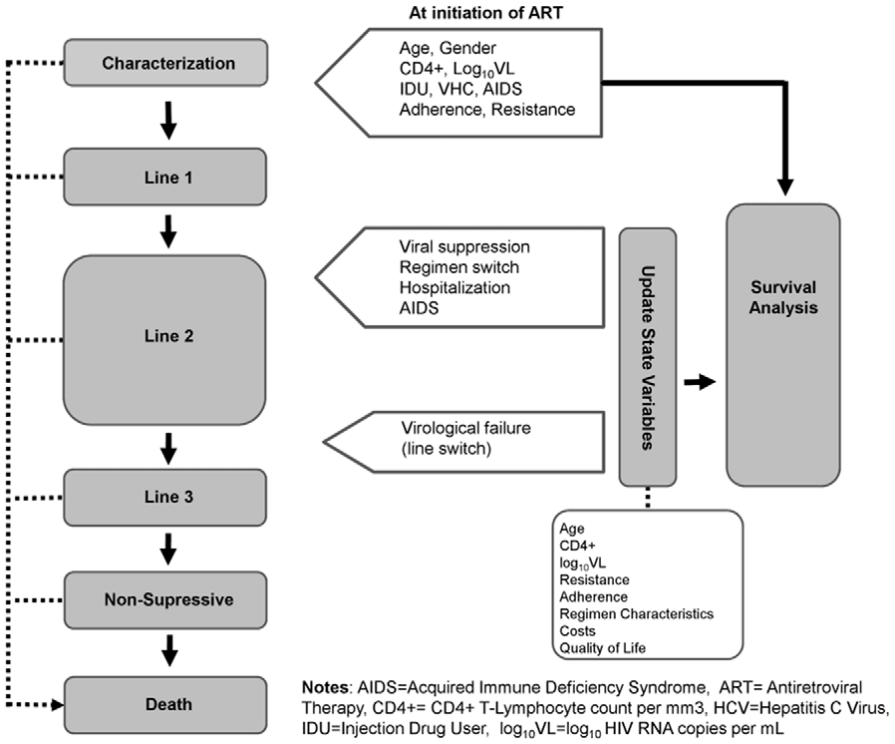
Discrete events microsimulation model diagram.

Figure 1 shows the structure of our model. In this model, an individual enters in the simulation when first-line ART is started. For each individual, the model captures initial characteristics such as age, gender, mode of transmission, employment status, co-infection with hepatitis C virus, AIDS diagnosis, HIV resistance level, adherence level, CD4+ cell count, viral load and age of death due to non-HIV related causes. Those characteristics are sampled from distributions that were previously fitted to cohort data. Once characteristics are assigned to the individual (and depending upon those), the model estimates the time to occurrence of the next clinical event. Clinical events considered were viral suppression (log_10_ of HIV RNA copies per mL (log_10_VL) <50 copies/mL), regimen switch without virological failure (any change in current regimen), line switch (implies virological failure), resistance development, hospitalization, AIDS-defining event, and death. At the occurrence of the event, evolving state variables (CD4+ cell count, viral load, adherence level, resistance level, AIDS status and regimen characteristics), costs and benefits are updated.

Regarding line switch, we assumed that the individual switches from first-line to second-line (and subsequently from second-line to third-line) if viral suppression is not reached within 12 months of line initiation or if virological failure (HIV-1 RNA level ≥50 copies/mL) is confirmed after viral suppression has been achieved. While a 6-month period is currently recommended, 12 months was the threshold used in clinical practice in the first half of the 2000 decade. Moreover, it should be noted that the 12 months period to reach viral suppression reflects 12 months to reach viral suppression and being tested, which depends on testing frequency. When entering in the third-line, the model assumes that the individual will stay on this same line as long as resistance is not in the highest class and regardless of the number of virological failures. Once the highest resistance class is reached, the individual starts non-suppressive therapy. This process is repeated until death occurs and the individual simulation is stopped.

Cost-effectiveness results are obtained by simulating the paths of one million individuals. The statistical analyses were performed in Stata 11®.

### Input Parameter Values

The model was parameterized with Portuguese observational data and when such was not available estimates available in the international literature were used. Portuguese observational data sources include three main databases with data at the patient level (The Communicable Diseases and Epidemiological Surveillance Center (CVEDT) database, the Egas Moniz Hospital (LVHEM) laboratory database and the Cascais Hospital (CHC) database) along with aggregate national data regarding deaths due to HIV/AIDS and inpatient cost per episode. Written consent for access to the databases was approved by the Ethics Committees of Hospital de Cascais, Centro Hospitalar de Lisboa Ocidental and Instituto Nacional de Saúde Dr. Ricardo Jorge, as required. The Ethics committees waived the need for informed consent from participants as the data were analyzed anonymously and retrospectively.

The CVEDT laboratory database is the national registry of HIV infected individuals (34,888 individuals in August 2009). The LVHEM database provides data on approximately 80% of HIV infected individuals tested for resistance in Portugal but it has a selection bias since the database tends to include those individuals with suspected resistance (5,456 individuals between 2001 and 2008) along with pre-ART resistance test results. This database was used to parameterize the initial resistance level (using pre-ART resistance tests) and lines 3 and 4 of the model.

The CHC database provides data on 1,306 HIV-1 infected individuals followed at one hospital unit in the vicinity of Lisbon, between 2001 and 2008. The data resulted directly from the electronic software used, on a daily basis, for patients follow-up. While not nationally representative, this was the most complete database available in the country providing socio-demographic information, clinical markers and resource consumption data. For the most part, CHC data was used to parameterize lines 1 and 2 of the model.

### Initial Patient Characteristics

The model simulates individual paths from the moment of ART initiation to death. At ART initiation the individual is characterized with respect to age, gender, employment status, hepatitis C virus (HCV) co-infection, AIDS diagnosis, mode of transmission, CD4+ cell count, viral load, adherence, resistance, and regimen characteristics (number of PIs in the regimen, daily frequency and number of pills per day).

Initial patient characteristics of the first ART regimen were mainly obtained from the subsample of ART naïve patients in the CHC database, who initiated treatment with 2NRTI+NNRTI (n = 158) or 2NRTI+IP/r (n = 159) during the follow-up period. Patients were not randomized to each group, nonetheless, no statistically significant difference was found with regard to gender (p = 0.751), median age (p = 0.649), co-infection with hepatitis C (p = 0.971), initial resistance to at least one drug (p = 0.792), median CD4 cell count (p = 0.500), median log_10_VL (p = 0.056), and median adherence measured by pharmacy refills (p = 0.327). Quantitative data was compared between groups using t-test for independent data, or Mann-Whitney if normality assumption was not accepted. For qualitative data, Chi-square test was used. A statistically significant difference was found regarding year of ART initiation with the 2NRTI+PI/r, on average, initiating 2 years later (2003 versus 2005, p<0.001). Reflecting the study period and clinical practice at the time, in that cohort, the most frequently used pair of NRTI was zidovudine/lamivudine, and lopinavir/ritonavir was the PI/r used in 73% of patients on PI-based regimen. Table 1 summarizes the distributions used to assign characteristics at ART initiation to the simulated individuals and corresponding data sources.

**Table 1:** Characteristics at ART initiation.

		2NRTI+NNRTI	2NRTI+PI/r	Distribution assumed	Source
Female		32.3%	34.0%	Bernoulli	CVEDT
Age, years		39 [33]; [46]	39 [33]; [50]	Table	Naïve at CHC
Employment statusa)		66%	66%	Bernoulli	CHC and Reis et al. 2007 [59]
HIV RNA, log_10_copies/mL		4.9 [4.3;5.4]	5.1 [4.3;5.5]	Table	Naïve at CHC
CD4+ cell count, cells/µL		234 [128;349]	219 [108;350]	Table	Naïve at CHC
HCV		29.8%	29.6%	Bernoulli	Naïve at CHC
Adherenceb), %		89 [71;98]	88 [73;96]	Table	Naïve at CHC
Year of ART initiation		2003	2005	Table	Naïve at CHC
NRTI pair	AZT+3TC	55%	52%		Naïve at CHC
	TDF+FTC	19%	23%		Naïve at CHC
	TDF+3TC	9%	9%		Naïve at CHC
	ABC+3TC	3%	11%		Naïve at CHC
	Others	15%	5%		Naïve at CHC
3rd agent	EFV	64%	0%		Naïve at CHC
	NVP	36%	0%		Naïve at CHC
	LPVr	0%	73%		Naïve at CHC
	Other PI/r	0%	27%		Naïve at CHC
Resistance level >1c), %		1.27	0.63		Naïve at CHC
Transmission groupa)	IDU	27.8%	27.8%	Table	
	Homosexual	13.7%	13.7%		CVEDT, 2004–2008
	Heterosexual	57.5%	57.5%		
	Other	1.0%	1.0%		
AIDS		32.2%	32.2%	Bernoulli	CVEDT/Naïve at CHC

Values presented in initial characteristics are median with interquartile range in square brackets [IQR] or mean or percentage (number followed by %).

a) Assumed identical among groups.

b) Adherence measured by pharmacy refills in first regimen.

c) Inverted Genotypic Sensitivity Score based on REGA 8.0 Algorithm.

Abbreviations: AIDS = Acquired immune deficiency syndrome; ART = Antiretroviral therapy; CD4+ = CD4+ T-Lymphocyte count per µl; CHC = Centro Hospitalar de Cascais; CVEDT = Communicable Diseases and Epidemiological Surveillance Center; HIV = Human Immunodeficiency Virus; HVC = Hepatitis C-Virus; IDU = Injection Drug User; LVHEM = Egas Moniz Hospital Virology Laboratory - Western Lisbon Hospital Center; NNRTI = Non-nucleoside Reverse Transcriptase Inhibitor; NRTI = Nucleoside Reverse Transcriptase Inhibitor; PI = Protease Inhibitors; PI/r = Boosted Protease Inhibitor; IQR = Interquartile range; RNA = Ribonucleic acid; AZT = zidovudine; 3TC = lamivudine; FTC = emtricitabine; TDF = tenofovir; ABC = abacavir; NVP = nevirapine; EFV = efavirenz; LPV/r = lopinavir plus ritonavir.

### Evolving State Variables

The model assumes that most of the initial characteristics will evolve over the lifetime of the individual being updated upon event occurrence and thereby influencing (together with cumulative history of events) the distribution of time to next event. Employment status, HCV co-infection, mode of transmission and gender are assumed constant over time due to either being immutable or due to lack of information in the data sources used.

Based on individual-level data from CHC database, the average monthly variation of CD4+ and log_10_VL was estimated by weighted non-linear least squares regression conditional on therapy line. In the estimation, we used a logarithmic function with line specific parameters to reflect the fact that CD4+ and log_10_VL evolution may not be linear (for example, CD4+ may increase more rapidly first and tend to a plateau afterwards) and may differ according to the accumulated number of virological failures. For modeling purposes, if the individual does not respond to the new regimen, log_10_VL will continue the estimated path until virological failure is declared due to lack of response. If viral load suppression is achieved, log_10_VL will decrease instantaneously and remain constant until a rebound occurs. The log_10_VL rebound value is sampled from a Uniform distribution obtained from the CHC database.

In this analysis, pharmacy refills were used to quantify adherence. Our estimate of adherence is the percentage of days, in each regimen, the individual could not have had medication in his/her possession - given the quantities dispensed, the dates of refill and accounting for the fact that often individuals will refill before the stock has gone down to zero. In order to model changes in adherence level over time, and given the fact that adherence is likely to depend upon individual and ART regimen characteristics, a generalized linear model with a binomial family and a Logit link [25], [26] was used to estimate the relationship between adherence and relevant predictors available in the CHC database (The last column of Table 2 provides estimates of the marginal effect of each covariate).

**Table 2:** Estimated Marginal Covariate Effect on Median Time to Clinical Events, Monthly ART and non-ART Outpatient Costs and Adherence Level.

		Time to clinical eventa)								Monthly costb)		Adherence levelc)
		Viral suppression (line 1 and 2)	Virological failure (line 1 and 2)	Virological failure (line 3)	Switch without virological failure (all lines)	First resistance	Resistance class switch	Hospitalization	Death	ART	Non-ART	
Female (male is reference)		−0.554	**7.653**	0.643	−2.702	8.590	−0.486	11.149	**1523**	−0.029	0.046	1.4%
Age, years		−**0.111**	0.559	**−0.080**	**−**0.079	**−5.180**	**−0.555**	−**2.502**	−**153**	0.0001	0.0001	0.1%
HIV RNA, log_10_copies/mL		**0.951**	−**19.563**	−**1.550**	**1.321**	−**84.450**	−**3.249**	−**18.216**		−**0.030**	**0.028**	
CD4+ cell count, cells/µL		0.000	**0.026**		0.003			**0.146**		**−0.0001**	**−0.0001**	
HCV		−**1.680**	10.094		−**5.223**	−26.000		−32.008		−0.015	0.020	−1.0%
Adherenced), %		−**0.062**	0.184		**−0.940**	**2.910**		0.424			**0.010**	
Year of ART initiation		0.022	2.448	−**5.188**	−1.189	−22.290	**0.020**	1.295		**0.035**	**0.042**	
Number of PIs in regimen		0.297	−**6.120**	−0.618	−**5.678**	−33.890	**6.850**	−3.453				−1.2%
Resistance level		−**0.847**	−1.894		0.269			−7.927		**0.039**	**0.021**	
Regimen number			−**8.963**	0.239	**1.071**	3.990	−**6.310**					−1.6%
Line (1 is reference)	2	0.485	−10.658		−2.836			−**34.500**		0.028	0.0001	−1.7%
	3				−1.444			−**64.061**				−3.1%
Previous virological failures (0 is reference)	1									0.028	0.0001	
	>1									**0.307**	**0.166**	
Resistance classe) (<1 is reference)	1≤R<5			**−6.225**								
	5≤R<10			−**7.348**								
	R≥10			−**7.021**								
Year of diagnosis									**660**			
Transmission (hetero is reference)	IDU								−**3692**			
	Homosexual								−**900**			
	Other								−**2300**			
AIDS (non-AIDS is reference)									−**18143**			
Shape parameter of Weibull distribution (p)		1.030	**1.447**	**1.873**	**1.499**	**1.501**	**1.524**	**0.733**	**(stratified by AIDS status)**			
Number of doses/day (2 is reference)	1											1.8%
	3											−2.5%
Number of pills/day												0.001%
ART duration, years												−0.01%
Monthly ART cost												0.001%

a) Marginal effects of covariates used in the adjustment of a Weibull distribution to time to clinical events.

b) Marginal effects of covariates used in a generalized linear model with a Gamma distribution with Log link function to estimate ART/non-ART costs.

c) Marginal effects of covariates used in a generalized linear model with a Binomial distribution with Logit link function to estimate adherence level.

d) Adherence measured by pharmacy refills.

e) Inverted Genotypic Sensitivity Score based on REGA 8.0 Algorithm.

Significant covariates (p<0.05) are in bold.

Abbreviations: AIDS = Acquired immune deficiency syndrome; ART = Antiretroviral therapy; CD4+ = CD4+ T-Lymphocyte count per µl; CHC = Centro Hospitalar de Cascais; CVEDT = Communicable Diseases and Epidemiological Surveillance Center; HIV = Human Immunodeficiency Virus; HVC = Hepatitis C-Virus; IDU = Injection Drug User; LVHEM = Egas Moniz Hospital Virology Laboratory - Western Lisbon Hospital Center; NNRTI = Non-nucleoside Reverse Transcriptase Inhibitor; NRTI = Nucleoside Reverse Transcriptase Inhibitor; PI = Protease Inhibitors; PI/r = Boosted Protease Inhibitor; IQR = Interquartile range; RNA = Ribonucleic acid.

Resistance level was defined as the sum of the inverted Genotypic Sensitivity Score obtained from the REGA 8.0 algorithm, after inverting this score (which measures sensitivity to the drugs) to reflect rather the resistance to the drugs. The resistance score was grouped in four classes (R<1, 1≤R<5, 5≤R<10, 10<R≤25) and it was assumed that individuals could move only to an adjacent resistance class. When the next event occurring to the individual corresponds to a resistance development, the individual moves to the following resistance class and a new resistance level is assigned sampled from a Uniform distribution within that class.

The simulated individuals may have been diagnosed with AIDS at ART initiation. If not, upon the occurrence of the first AIDS-defining event or a CD4+ <200 cells/mm^3^, the individual is permanently classified as having AIDS. Subsequent AIDS-defining events may occur with impact on both costs and effectiveness indicators.

Regimen characteristics (number of PIs, frequency of daily dosing and total number of pills per day) are updated on occurrence of line switch or regimen switch without virological fail. Upon event occurrence, a random draw from a Table distribution (conditional on line number) will determine the new value for each of the three variables.

### Time to Clinical Event

In simulation survival analysis was used to link time to event to individual characteristics, accumulated history and regimen characteristics depending on data availability. Weibull distributions were considered in the parameterization of survival curves for each clinical event (covariate marginal effects are presented in Table 2 in the columns under the heading “Time to clinical event”). Following Barton *et al.* [21], the conditional distribution was used to sample time to next event. Selection of the next event to occur also uses the methodology adopted in Barton *et al.* [21].

#### a) Virological suppression

Since time to virological suppression is expected to vary according disease stage, survival curves were fitted for line 1/line 2 separately from line 3 on which patients are at a symptomatic stage of the infection. A Weibull distribution was fitted to CHC sample for line 1 and line 2 whereas data from two clinical trials [27], [28] was considered for line 3 (not conditioned to individual characteristics or past history) due to unavailability of national data.

#### b) Regimen switch without virological failure

Regimen switch is defined as a change of at least one of the drugs in the actual regimen, adding a new drug or withdrawing a current one. The CHC database was used to adjust a distribution for time to regimen switch independently from the treatment line. Since the reason for switching was not available, it was assumed that the cause of regimen switch without virological failure was proportional to that observed in the Swiss Cohort [29] (54% of regimen switches due to toxic effects).

#### c) Line switch – virological failure

A line switch event is defined as an ART regimen modification resulting from a virological failure (defined as a confirmed HIV-1 RNA level ≥50 copies per milliliter after viral suppression has been achieved or unreached viral suppression 6 months after regimen initiation). Virological failure may occur for several reasons but since those were not available, a unique event was considered regardless of the cause. A Weibull distribution was fitted for time to line switch for line 1 and line 2 using the CHC sample whereas LVHEM sample was used to model time to event regarding line 3.

#### d) Resistance development

The information to parameterize the model with respect to time to first resistance development was obtained from the CHC sample. Time to resistance class switch once the first resistance has developed was estimated on the LVHEM database since it provides a sample of individuals who have some positive level of resistance (and 76% are resistant to at least two drugs). LVHEM database does not however contain information on adherence and consequently the relationship between adherence and resistance could not be estimated. Predicted median time to resistance was much longer for the first resistance than for when some level of resistance had already been developed (28 years versus 7 years) likely reflecting cross-resistance and cumulative exposure.

When the individual reaches non-suppressive therapy, he will be in the highest class of resistance. In order to account for the fact that resistance will eventually increase within class 4, we have used the model in Table 2 (column 6 of the “Time to clinical event” set of columns - heading “Resistance class switch”) to sample time to resistance increase within line 4. Upon such event occurrence, the new resistance level is assumed to be the maximum between the current level of resistance and a random draw from a Triangular distribution fitted on resistance levels above 10 among individuals in line 4 of the LVHEM sample.

#### e) Hospitalization

Data in CHC database was considered for modeling time to hospitalization. The discharge date was the only information available and therefore was used as a proxy for the date of the hospitalization. Hospitalizations occurring immediately before ART were excluded since those were considered to be related with the absence of ART.

#### f) AIDS-defining event

Due to lack of information on national databases, time to AIDS-defining event was assumed to follow the Weibull proportional hazards model stratified on CD4+ and transmission risk published by May et al. [30], [31] using data from the ART Cohort Collaboration and considering new AIDS defining disease or death from any causes as a composite endpoint. Given this limitation, we parameterized the model under the assumption that all observed deaths were preceded by an AIDS event.

#### g) Death

Time to death among HIV infected individuals was adjusted to a Weibull distribution stratified by disease stage (AIDS/non-AIDS) using data from CVEDT database. In order to account for the fact that mortality due to age may not yet be correctly reflected in the CVEDT database (due to 30 years of the epidemics and the young age of those infected.), the model assumes that the estimates provided by the CVEDT refer to HIV related deaths whereas deaths due to other causes were included as a competing risk. Parameterization of the model, regarding death due to other causes was based on Portuguese general population mortality rates in the 2006–2008 period published by the National Institute of Statistics database [32]. The sampling process followed Barton et al. [21].

While, in the model a parametric survival analysis approach was used, it is worth nothing that in the CHC naive sample, a statistical difference was found in Kaplan-Meier curves of time to first regimen switch without virological failure with medians (95% confidence interval) of 6.7 (3.5–7.1) and 2.7 (2.3–3.7) years for 2NRTI+NNRTI and 2NRTI+PI/r (HR: 1.65, 95% CI: 1.14–2.42, Log-rank test: p = 0.008), respectively. No statistically significant difference was found between groups in terms of time to viral suppression (p = 0.072), virological failure (p = 0.579), first resistance development (p = 0.561).

### Costs

Unitary drug costs were extracted from the Catalogue of Health Procurement [33] in December 2009 and when necessary those published at the National Authority of Medicines and Health Products IP website. The other health resources were valued at the prices published by the Portuguese Health Ministry [34]. All costs were expressed in 2009 euros.

First regimen ART and non-ART costs were obtained from the subsample of ART naive patients in the CHC database. A statistically significant difference was obtained for the median [interquartile range] of first regimen monthly cost (2NRTI+NNRTI: 613€ [525€;797€]; 2NRTI+PI/r: 1057€ [853€;1057€], p<0.001) and these estimates were used in the model.

In line with the approach followed to estimate effectiveness, the CHC sample was the main source for ART resource consumption in first and second line and LVHEM sample for third line and non-suppressive therapy.

A monthly ART cost was estimated based on individual characteristics through a generalized linear model (column 3 from the right in Table 2). In non-suppressive therapy, a daily ART cost is assigned to each individual through a Lognormal distribution fitted on the daily regimen costs of individuals at class 4 of resistance in the LVHEM sample.

Outpatient costs included non-ART medication provided by the hospital, physician appointments, exams and laboratory tests. Reflecting clinical practice, consumption of these resources was taken directly from CHC database. Since such information was not available in any of the remaining databases, the CHC database was used to estimate non-ART costs for all therapy lines. Estimates of first line outpatient monthly cost were obtained from the subsample of ART naive patients (2NRTI+NNRTI: median 63€ [24€;106€]; 2NRTI+PI/r: 98€ [41€;137€], p = 0.008). For the remaining therapy lines, a monthly non-ART cost conditional on individual characteristics was estimated using a generalized linear model with a Gamma distribution and a log link function (column 2 from the right in Table 2).

Health resource consumption upon regimen switch with and without virological failure was calculated based on the set of procedures recommended by the Portuguese Guidelines for HIV/AIDS infection [7], with a total cost of 582€ and 495€, respectively.

The cost of adverse events was obtained from the literature [35] considering the average direct cost of a moderate or severe intolerance episode (1,126€ at 2005 prices then converted for 2009 prices using the inflation rates reported by the Statistical Division Database of the United Nations Economic Commission for Europe [36]).

Costs of AIDS events and hospitalizations were obtained from the national database of inpatient episodes [ ]. The cost of an AIDS event was assumed to be equivalent to the average cost of episodes with International Classification of Diseases 9-Clinical modification code 042 (“Human immunodeficiency virus [HIV] disease” which includes AIDS and symptomatic infection) (average cost: 4,765€; average length of stay: 19.3 days) and the cost of a hospitalization to be the average cost of the remaining HIV related code episodes (average cost: 4,742€; average length of stay: 14.9 days).

The present cost-effectiveness analysis considers HIV-1 infected treatment naïve adult individuals. In accordance with the Portuguese guidelines, both costs and effectiveness were discounted at 5% [37] and assessed over a lifetime horizon. The analysis was conducted from the Portuguese NHS and included only direct medical costs.

### Quality of Life

Life-years were converted in quality-adjusted life years (QALYs) applying published utility estimates [38], derived from a sample of 21.000 clinical trial patients using preferences of the population in the United Kingdom based on the EQ-5D quality of life instrument and stratified by CD4+ count and virological load.

Cost-effectiveness analysis was performed considering the total costs of each treatment option along with two effectiveness indicators were used: life-years and QALYs. Incremental cost-effectiveness ratios included the incremental cost per life-year gained and per QALY gained.

## Results

### Microsimulation Cost-effectiveness Results

Microsimulation outputs were obtained by simulating 1 million individuals. Clinical outcomes predicted by the model are described in Table 3. In line with the Kaplan-Meier estimates of time to event discussed previously, clinical outcomes predicted by the model for ART initiation with 2NRTI+NNRTI and 2NRTI+PI/r did not differ significantly except for the average number of regimen switches over the life time of the individual. Our results indicate that initiating ART with 2NRTI+NNRTI will reduce the total number of regimen switches over the individual lifetime in 17%.

**Table 3:** Clinical outcomes from the model.

		2NRTI+NNRTI	2NRTI+PIr	Percent Difference
Months without viral suppressiona)	Line 1	7.55	7.55	0.0%
	Line 2	7.17	7.25	**−**1.2%
	Line 3	17.95	17.66	1.7%
% attaining viral suppression	Line 1	68%	68%	**−**0.1%
	Line 2	55%	55%	**−**0.4%
	Line 3	46%	46%	0.0%
Variation in CD4+ cell count, cells/µL	Line 1	265	264	0.1%
	Line 2	180	181	**−**0.4%
	Line 3	101	102	**−**0.2%
	Non-suppressive	**−**49	**−**50	**−**1.5%
% reaching each line	Line 2	0.76	0.75	1.8%
	Line 3	0.49	0.48	0.6%
	Non-suppressive	0.33	0.32	4.0%
Life time events	Regimen switch	4.16	5.02	**−**17.2%
	Hospitalization	4.55	4.70	**−**3.1%
	Virological failure	3.53	3.50	0.7%
	Failure after suppression	1.53	1.52	0.4%
	Suppression not achieved	2.00	1.98	1.0%

One million individuals simulated. Values undiscounted.

a) Within patients who reached virological suppression.

Abbreviations: CD4+ = CD4+ T-Lymphocyte count per µl.

Table 4 gives an overview of the base-case results with costs and effects discounted at 5% and an additional scenario without discounting. In the base-case analysis, the mean lifetime cost of an individual initiating ART with a 2NRTI+NNRTI regimen was 172.742€ in comparison with 192.315€ for ART initiation with a 2NRTI+PI/r regimen. Therefore, initiating ART with 2NRTI+NN results in savings of 19.573€ over the lifetime of the individual (about 1.250€ per year) or a cost reduction of 10%. These savings occur both in ART (**−**15.753€) and non-ART costs (**−**3.821€). The most significant cost differences are seen in first line since treatment in subsequent lines is only slightly conditional on first line ART in each group (through the number of PIs and the number of accumulated different regimens). In both regimens, ART is the cost-driver representing 79% of the total cost. This cost distribution reflects the fact that progress in ART has improved overall health and thus decreased the frequency and severity of opportunistic infections/AIDS-defining episodes.

**Table 4:** Costs and Life Years Microsimulation Results.

	Discounted (5%)		Undiscounted	
	2NRTI+NNRTI	2NRTI+PIr	2NRTI+NNRTI	2NRTI+PIr
ART costs	135.406 €	151.158 €	273.294 €	295.529 €
Non-ART costs	37.336 €	41.157 €	73.998 €	79.941 €
Total costs	172.742 €	192.315 €	347.292 €	375.470 €
Δ costs	**−**19.573 €		**−**28.178 €	
Life years	15.69	15.57	24.35	24.14
Δ life years	0.12		0.21	
QALYs	11.84	11.70	22.84	22.43
Δ QALYs	0.14		0.41	
ICER (€/LY)	Dominant		Dominant	
ICER (€/QALY)	Dominant		Dominant	

One million individuals simulated.

Abbreviations: ART = Antiretroviral therapy; CD4+ = CD4+ T-Lymphocyte count per µl; ICER = Incremental cost-effectiveness ratio; LY = Life Years; QALY = Quality adjusted life year.

Annual non-ART costs increases along the therapy lines indicating a more intense consumption of health resources as the disease progresses. Except for non-suppressive therapy, annual ART-costs are also increasing in therapy line reflecting the need for more complex and expensive drugs as resistance develops. Annual ART costs in non-suppressive therapy are estimated to be lower than those for third line which is an unrealistic result given the currently available option for highly resistant patients (data not shown). This result is a consequence of the 2001–2008 period of analysis in the datasets.

As presented in Table 4, the model predicts a life expectancy of about 15.69 and 15.57 years in individuals starting ART with 2NRTI+NNRTI and 2NRTI+PI/r, respectively (difference of approximately 1.2 months). When adjusting for quality of life, a total of 11.84 and 11.70 QALYs are expected when selecting, respectively, NNRTI and PI/r as the third agent in the initial ART regimen. Thus, initiating with 2NRTI+NNRTI results in a slight health benefit of 0.14 QALYs gained (1.7 months). Without discounting, a cost-saving of 28.278€ and a gain of 0.41 QALYs is expected per individual initiating with 2NRTI+NNRTI.

According to this analysis, initiating ART with 2NRTI+NNRTI results in lower total health care costs over the life span of the individual and such gain is obtained with a marginal increase in effectiveness as measured by the estimated number of QALYs. Selecting NNRTI as the third agent class is thus a dominant strategy when compared to initiating ART with 2NRTI+PI/r.

### Probabilistic Sensitivity Analysis

In the context of microsimulation models it is relevant to differentiate two categories of uncertainty: the first-order which is associated with the variability among individuals and the second-order related with parameter uncertainty. The first is reflected in the microsimulation outputs whereas the second was incorporated in the current probabilistic sensitivity analysis, which is performed by allowing all input parameters to vary simultaneously. The acceptability curve was obtained by simulating 50.000 individuals for 133 different set of combinations of parameter values sampled from appropriate probability distributions via Monte Carlo simulation. The ranges and distributions used in the uncertainty analyses were derived from the estimated standard errors (when related to parameters obtained from databases) or published sources (when parameters where drawn from the international literature).

**Figure 2:**
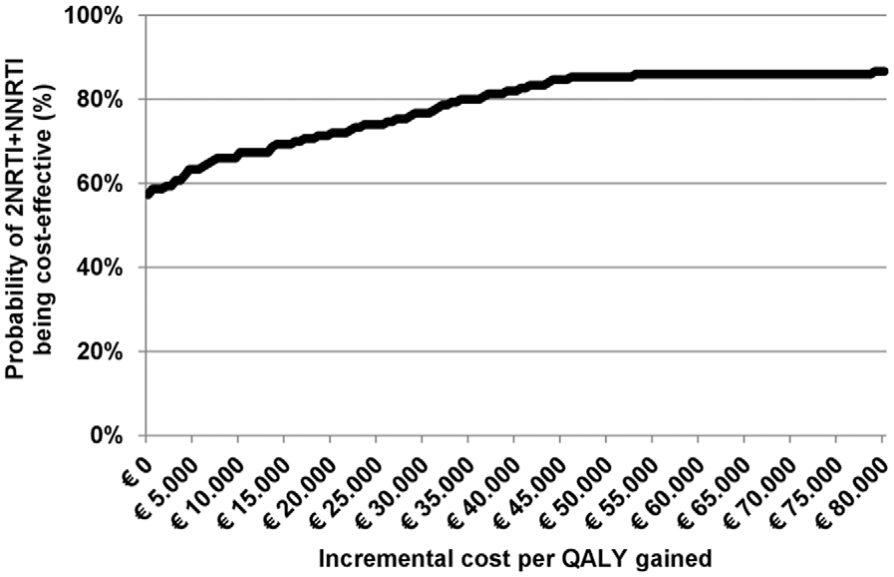
Probabilistic sensitivity analysis: cost-effectiveness acceptability curve for 2NRTI+NNRTI compared with 2NRTI+IP/r as initial antiretroviral therapy.

Given the estimated small difference regarding the total number of QALYs in the two strategies, it is of special interest to evaluate the impact of the assumed parameter values on the base-case results. In the probabilistic analysis 57% of the simulations yielded 2NRTI+NNRTI as a dominant strategy. The acceptability curve (Figure 2) shows that for a willingness-to-pay threshold of 30.000€ per QALY, the probability of 2NRTI+NNRTI being cost-effective is 85%.

## Discussion

In the current cost containment environment, there exists an urgent need to identify strategies that will reduce costs without compromising the health benefits ART has generated. Economic evaluation, by providing information on the cost per QALY gained, provides policy makers and health care professionals an efficiency oriented tool to support decisions.

Two cost-effectiveness analyses have been performed comparing NNRTI to PI/r as a third agent in the initial treatment of HIV-1 infected individuals [15]. Although performed in very distinct settings (Beck et al. [39] in the United Kingdom, Walensky et al. [40] in Côte d’Ivoire), both concluded that initiation with 2NRTI+NNRTI, when clinically viable, was a dominant option when compared to 2NRTI+PI/r. More specifically, in the work by Beck et al. 2NRTI+NRTI is a dominant strategy in patients with CD4+ >200 cell/µL. In patients with CD4+<200 cell/µL 2NRTI+PI/r yields higher effectiveness but with an incremental cost-effectiveness ratio above that considered acceptable by the National Institute for Health and Clinical Excellence (NICE). Walensky et al. find 2NRTI+NNRTI to be a dominant strategy even in areas of high prevalence of NNRTI resistance (up to 76%). In this context, we have found it relevant to evaluate this potentially dominant strategy in the Portuguese context and indeed our results are in line with those available in the literature.

Although a model represents a simplification of reality, it should be able to reproduce the main aspects under consideration. In our simulation, the percentage of individuals attaining viral suppression in the first line (68%) was lower than in clinical trials like in Study 934 [41] where 70% reached this endpoint in zidovudine arm and 80% in tenofovir at week 48. This is likely to reflect the difference between efficacy (clinical trial context) and effectiveness (daily basis clinical practice setting).

The percentage of individuals who reaches viral suppression decreases with the number of previous therapy failures and is in line with clinical trial results of naïve and experience patients. Model predictions for the percentage of experienced individuals reaching viral suppression were similar to those observed in clinical trials [27]. The estimated average of 7.6 months to viral suppression matches the input cohort data. Greenbaum et al. [42] estimated a median time to first undetectable HIV-1 RNA level of 5.4 months, which is not directly comparable. Moreover, the event modeled is “HIV RNA<50 copies/mL test result” which depends not only on ART efficacy but also on testing frequency. Moreover, the model predicts time to viral suppression to be identical in both groups but literature is contradictory in such comparison [42], [43].

CD4+ yearly increase was estimated to be 28, 24 and 21 cells/mm3 in first, second and third line, respectively, which are similar to those estimated by Phillips et al. [44] on the EuroSIDA cohort whereas on non-suppressive therapy, CD4+ cell count is predicted to decrease at a rate of 17 cells/mm3 per year, which is slightly lower that the rate estimated by Plato collaboration [45].

According to the model, at ART failure, the viral load increases about 83% of the level at ART initiation (not shown). This value is close to the estimates reported in the literature where rebound viral load levels are about 10% less than the original levels [46]–[48].

The total number of regimen switches without virological failure (4.2 in the NNRTI group and 5.0 in PI/r group) is the variable where the highest percent difference between groups was found, what is in alignment with CHC sample (namely, the statistically significant difference found in the survival curves of such event) and clinical trials results [49].

The ART Cohort Collaboration [50], [51] estimated that life expectancy of HIV infected individuals at the age of 20 was about two-thirds of that in the general population in each country and that overall, at the age of 35, life expectancy is 31.7. In Portugal, according to INE [32] the general population life expectancy at the age of 39 is 39.5, thus two-thirds would be 26.6, which is close to the 24.3 years predicted by the model. The difference found is reasonable given the higher proportion of injection drug users and AIDS diagnosis found in our sample compared with the ART Cohort Collaboration study.

Of the 24 predicted years, the model estimates that an average of 9 years will be spent in first line. This value is identical to the predicted value obtained using a Markov model parameterized with the same CHC database [52] but the average time in first line with currently recommended regimens is likely to exceed 9 years. The estimated time in first line is significantly lower than that published by Beck et al. [39], but definition of end of line differs and results are not comparable.

We predicted a 5 months difference in the average time spent in first line in the two groups, favoring the 2NRTI+NNRTI option. Once again our results contrast with those of Beck et al. [39] but coincide with those of Geretti et al. [53] who uses a definition of failure identical to ours. As in available literature [54], [55], the predicted average number of years spent in each subsequent line is decreasing in line number.

The overall cost of treating an HIV infected individual was 375,470€ in the 2NRTI+PI/r group (7.5% less in 2NRT+NNRTI group) with 79% being related with ART acquisition. This proportion seems reasonable given the increasing weight of ART cost in the overall treatment cost of HIV, from 50% in 1996/97 (at the time HAART was introduced) to 70% in 2000/2001 (at the time PIs became available) [56].

The predicted average annual cost of ART in first line was 9,010€ in 2NRTI+NNRTI and 23% higher in 2NRTI+PI/r suggesting that the annual payment of 11,040€ per infected individual established by the Portuguese Government in the HIV financing program for ART naïve individuals covers first line ART costs, although not first line total costs.

Our analysis has limitations that should be considered when interpreting the results.

First, it is of relevance to highlight the fact that the present analysis is based on cohort observational data rather than clinical trials. Observational cohort data highlights reality beyond clinical trials and is of relevance to correct factors that are overestimated in clinical trial settings, such as adherence. Nonetheless, utilization of observational cohort data has implications on results which should be taken into consideration. Our results indicate that, on average, a patient found (according to clinical decision) more suitable to initiate ART with a NNRTI based regime, will have a lower total lifetime cost than a patient found (again according to clinical decision) more suitable to initiate ART with a PI/r based regime. This result does not imply that, on average, 19,573€ could be saved per patient initiated with a NNRTI based regimen instead of a PI/r based regimen.

Second, while all efforts have been made to parameterize the model so as to reflect Portuguese clinical practice, given the unavailability of a national database complete enough for estimation of all parameters of interest, several databases were used. Moreover, due to sample size restrictions, the complete observation period had to be considered for estimation reflecting clinical practice from 2001 to 2008. One implication is that our analysis (like those of Beck et al. [39] and Walensky et al. [40]) was performed using data from a cohort of individuals whose regimens included antiretroviral drugs no longer recommended as a first choice.

Third, we have considered a single resistance score and, consequently, a single resistance event. There is evidence in literature suggesting that the likelihood of resistance development is drug specific, or at least, drug class specific [57]. Separately modeling resistance to each class has the important advantage of allowing for the selection of the future ART regimens to be based on resistance history. Both Braithwaite et al. [58] and Johnston et al. [17] followed that path.

Forth, while CD4 and viral load continue to be the main markers for disease progression, in order to account for long-term safety, other clinical parameters will need to be modeled (lipid profile, creatinine, bone density, etc.) and its consequences quantified.
